# New Liquid Crystal Assemblies Based on Cyano-Hydrogen Bonding Interactions

**DOI:** 10.3389/fchem.2021.679885

**Published:** 2021-06-04

**Authors:** Mohamed Hagar, Hoda A. Ahmed, Rua B. Alnoman, Mariusz Jaremko, Abdul-Hamid Emwas, Salim Sioud, Khulood A. Abu Al-Ola

**Affiliations:** ^1^ College of Sciences, Chemistry Department, Yanbu, Taibah University, Yanbu, Saudi Arabia; ^2^ Faculty of Science, Chemistry Department, Alexandria University, Alexandria, Egypt; ^3^ Department of Chemistry, Faculty of Science, Cairo University, Cairo, Egypt; ^4^ King Abdullah University of Science and Technology (KAUST), Biological and Environmental Sciences and Engineering Division (BESE), Thuwal, Saudi Arabia; ^5^ King Abdullah University of Science and Technology, Core Labs, Thuwal, Saudi Arabia; ^6^ King Abdullah University of Science and Technology, Analytical Chemistry Core Lab, Thuwal, Saudi Arabia; ^7^ College of Sciences, Chemistry Department, Madina Monawara, Taibah University, Al-Madina, Saudi Arabia

**Keywords:** cyano-hydrogen bonding interactions, supramolecular liquid crystals, molecular geometry, DFT – density functional theory, cyano-assemblies

## Abstract

A new selection of supramolecular liquid crystal complexes based on complementary molecules formed via hydrogen-bonding interactions is reported. All prepared complexes were prepared from 4-n-alkoxybenzoic acid (An) and N-4-cyanobenzylidene-4-n-(hexyloxy)benzenamine (I). FT-IR, temperature gradient NMR, Mass Spectrometer and Chromatography spectroscopy were carried out to confirm the -CN and −COOH H-bonded complexation by observing their Fermi-bands and the effects of the 1H-NMR signals as well as its elution signal from HPLC. Moreover, binary phase diagrams were established for further confirmation. All formed complexes (I/An) were studied by the use of differential scanning calorimetry and their phase properties were validated through the use of polarized optical microscopy Results of mesomorphic characterization revealed that all presented complexes exhibited enantiotropic mesophases and their type was dependent on the terminal lengths of alkoxy chains. Also, the mesomorphic temperature ranges decreased in the order I/A6 > I/A8 > I/A10 > I/A16 with linear dependency on the chain length. Finally, the density functional theory computational modeling has been carried out to explain the experimental findings. The relation between the dimensional parameters was established to show the effect of the aspect ratio on the mesophase range and stability. The normalized entropy of the clearing transitions (∆S/R) was calculated to illustrate the molecular interaction enhancements with the chain lengths.

## Introduction

Supramolecular H-bonded liquid crystal complexes (SMHBLCs), which have been known for decades, began receiving more attention in the middle of the 20th Century (Fairhurst et al., 1998). Such interactions might be H-bonding (Paleos and Tsiourvas, 2001; Kato et al., 2006; Demus et al., 2011), or halogen bonding (Nguyen et al., 2004; Metrangolo et al., 2006; Präsang et al., 2008; Wang et al., 2018; Alaasar et al., 2019; Saccone and Catalano, 2019) with both having the advantage of a formed liquid crystalline compound compared to covalent-bonding interactions. In general, hydrogen bonds are non-covalent, directional interactions between H-bond donor and an acceptor such as O or N atoms. The hydrogen bonds are either intramolecular, when they are in the same molecule, or intermolecular, if the interacting groups belong to different molecules. Hydrogen bond has essential role in the formation of mesophases in liquid crystals. Recently, various SMHBLCs were documented as different kinds of H-donors and H-acceptors that offer wide many structural shapes (Ahmed and Khushaim, 2020; Al-Mutabagani et al., 2020c). These included calamitic complexes (Naoum et al., 2008; Ahmed et al., 2019a), angular-shaped (Gimeno et al., 2004; Gimeno et al., 2008; Alaasar et al., 2011; Wang et al., 2015) polymeric architectures (Korkmaz et al., 2016), modular hierarchical (Pfletscher et al., 2016), nematogenic non-symmetric dimers (Korkmaz et al., 2016) or observing the twist-bend nematic mesophase (Walker et al., 2018) and supramolecular-polycatenars of chiral cubic-mesophases (Alaasar et al., 2016).

Of particular interest to us are new architectural materials with innovative shapes. (Yagai and Kitamura, 2008; Yeap et al., 2009; Hagar et al., 2019). These investigations have led to insights on the relationship between the experimental transitions and estimated computational results for SMHBCs. Many researchers (Paleos and Tsiourvas, 2001; Armstrong and Buggy, 2005; Tschierske, 2013; Goodby et al., 2014; Ahmed and Naoum, 2016; Ahmed et al., 2018; Ahmed et al., 2019c; Saccone et al., 2019; Tuchband et al., 2019; Martinez-Felipe et al., 2020; Walker et al., 2020) have analyzed new SMHBCs between their carboxylic acid and pyridine containing sections, with the intent to shed light on the menophase property of these new SMHBCs (Al-Mutabagani et al., 2020a). It was reported that the type of H-acceptor could impact the development of already existent properties, thus adding a new structural property to the liquid crystalline material (Al-Mutabagani et al., 2020a). Additionally, atom type and electronic nature are essential in the formation, the thermal stability, the type, and the mesomorphic ranges of the LCs phases (Cho et al., 2013; Paul et al., 2014; Alaasar et al., 2020; Blanke et al., 2020; Bryndal et al., 2020; Devadiga and Ahipa, 2020; Yilmaz Canli et al., 2020).

It was investigated and documented (Vijayalakshmi and Sastry, 2009) that, the phase behavior of 1:1 SMHBCs between 4-n-alkyl benzoic acids and 4-(4′-octyloxy benzylidene)-cyano aniline (Vijayalakshmi and Sastry, 2009). This study was resulted an enantiotropic liquid crystalline mixtures with induced smectic A (SmA) mesophase. The attached semi-flexible terminal chains lead to sufficient disorder and maintains the mesogenic cores at a slightly different positional order from the isotropic mesophase, thus influencing the formation of SmA phase. Another example (Kumar et al., 1999) of cyano H-bonding interactions is the supramolecular complexes between 4-n-alkoxybenzoic acids and 4-aminobenzonitrile. The intermolecular H-bonding interactions between the electron rich terminals CN and –COOH moieties leads to induced smectic G (SmG) phase. Moreover, the steric repulsive impacts of the terminal substituent are essential to stabilize the induced SmG phase (Kumar et al., 1999).

Recently, there are only a few reports regarding CN-based supramolecular hydrogen-bonding liquid crystal complexes (Paul et al., 2014; Chen et al., 2020; Meddeb et al., 2021). The aim of this work is investigate the preparation, mesomorphic, optical properties and structural parameters of newly synthesized supramolecular complexes (I/An) of CN based H-acceptor derivatives. Another aim of this study is to combine computational modeling of geometrical simulations with experimental findings for further studies regarding SMHBCs structural and physical properties. Herein we investigate the mesomorphic behavior of SMHBCs as well as their geometrical parameters by DFT simulation, and correlate the experimental data of the mesomorphic transitions behaviors with their calculated geometrical and thermal data values as continuing our interest (Al-Mutabagani et al., 2020b; Hagar et al., 2020; Nafee et al., 2020a; Ali et al., 2021; Almehmadi et al., 2021; Mohammed et al., 2021; Parveen et al., 2021) in conducting the experimental results with density functional theory (DFT) theoretical calculations.

## Experimental

**FIGURE 1:**
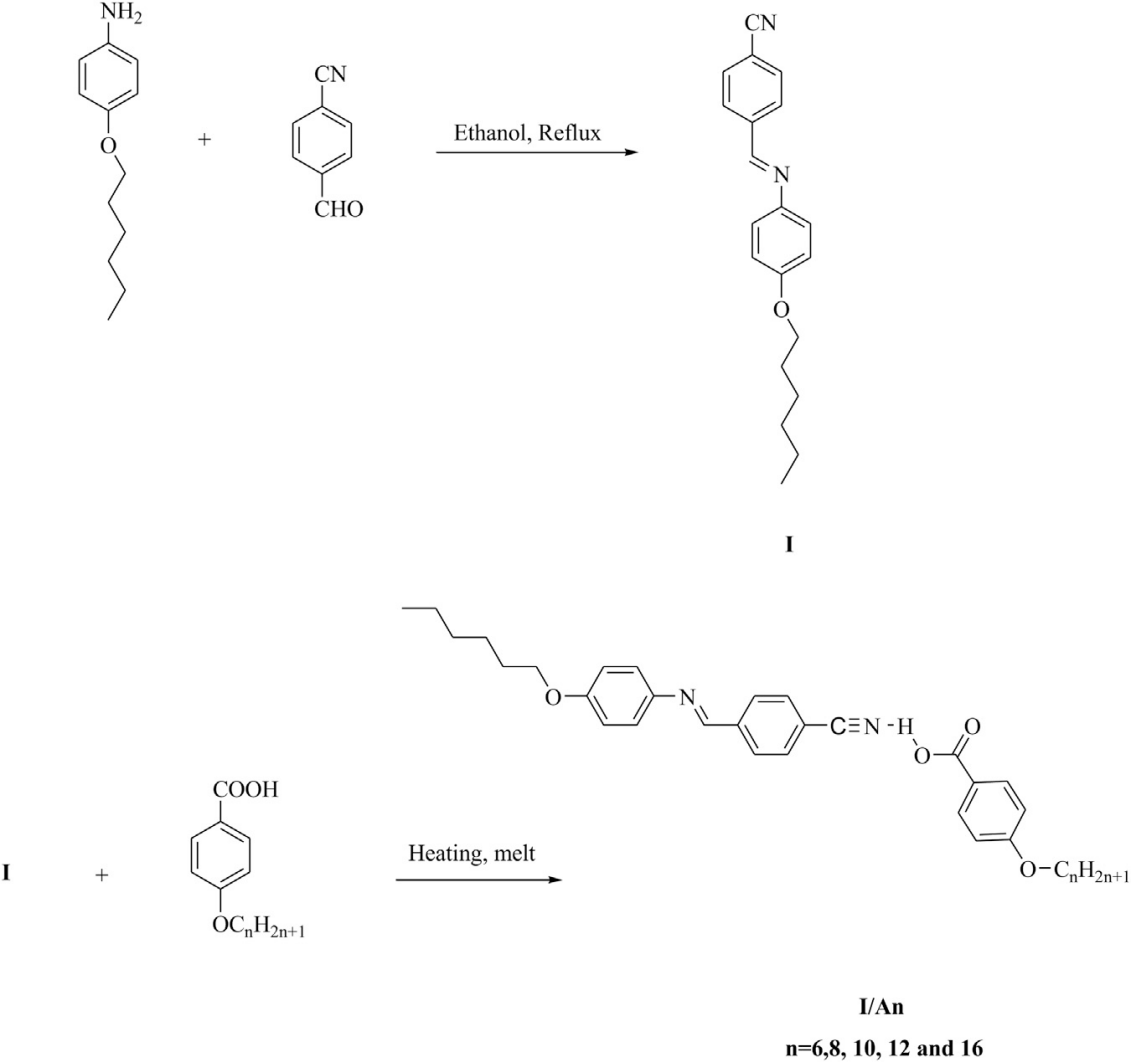
Synthesis of 1:1 SMHBCs I/An.

The CN derivative I and their SMHBC, I/An, were designed according to Figure 1:

### Preparation of Complexes, I/An

The CN derivative I was prepared according to the previous reported method (Ahmed et al., 2020). SMHBCs (I/An) were prepared by molar mixing of 1:1 M ratios of alkoxy benzoic acids (An) with changeable chain length from *n* = 6, 8, 10, 12, and 16 and the cyano Schiff base (I) with hexyloxy chain length. The mixture was melted with stirring till the intimate blend, then allowed to cool, Figure 1.

The NMR have been recoded for compound I/n8 that was prepared by dissolving in 600 µL of deuterated solvents DMSO-d6 inside a 3 ml glass vial, then vigorously vortexed until completely dissolved. 500 μL was transferred to 5 mm NMR tubes. A Bruker 600 NMR spectrometer (Bruker BioSpin, Rheinstetten, Germany) operating at 600.13 MHz for proton equipped with a triple resonance probe was used to record all NMR spectra. The ^1^H NMR spectrum was recorded by collecting 64 scans with a recycle delay time of 10 s, using one pulse sequence through a standard (zg) program from the Bruker pulse library. The ^13^C NMR spectra were recoded using the reported methods and parameters (Berger and Braun, 2004). Chemical shifts were corrected using the TMS signal at 0.0 ppm as an internal chemical shift.

## Results and Discussion

### Characterization of the Complex

#### FT-IR Confirmations

**FIGURE 2:**
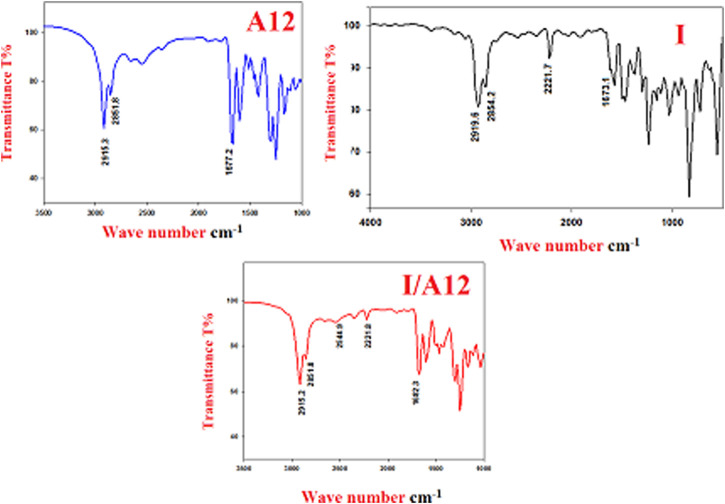
FT-IR 1:1 supramolecular H-bonded complexes (I/A12).

One of the main documented evidences of SMHBC formation is the OH-Fermi vibrational stretching bands (Saunders and Hyne, 1958; Lam et al., 2016; Martinez-Felipe et al., 2016; Hu et al., 2017; Pothoczki et al., 2020). The H-bonded OH functional group has three Fermi-resonance stretching vibration peaks (A-, B-, and C-type), indicating SMHBC interactions. The lye at the C–H vibrational frequency of 2,915–2,851 cm^−1^ was the peak of the A-type Fermi band of the complex **I/A12**. The peak at 2,544 cm^−1^ (I/A12) is also recognized as the B-type of the stretching vibration in-plane bending of the O–H group. However, the 1920 cm^−1^ Fermi band of the C-type is attributed to the interaction between the overtone of the torsional and the essential influences of the OH stretching vibration Figure 2.

#### NMR Confirmations

**FIGURE 3:**
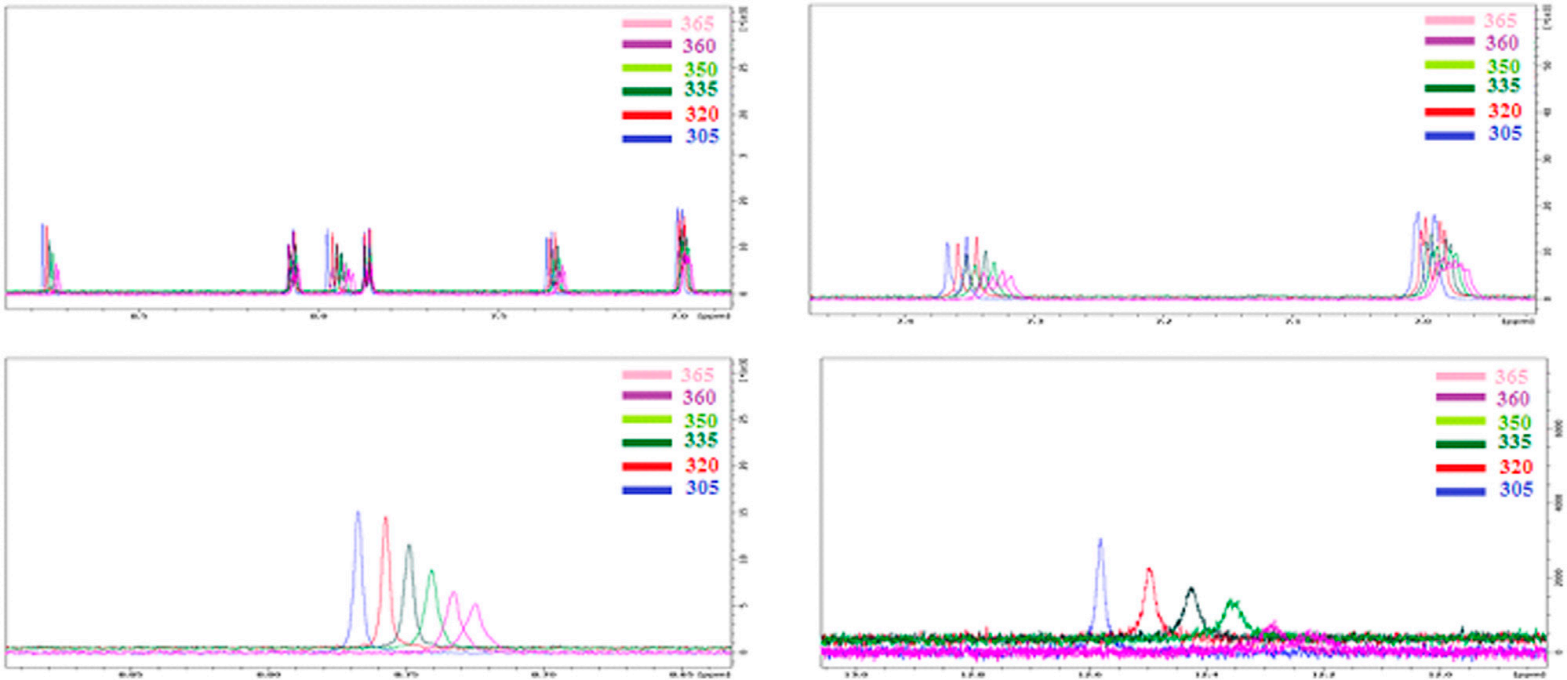
NMR 1:1 supramolecular *H*-bonded complexes (I/A8) with temperature gradient, T = 305–365 K.

The NMR results at temperature gradient showed a significant impact on characteristic signals. As shown from Figure 3, the gradient thermal heating of the DMSO solution of the prepared SMHBC I/A8 show a shift in the peaks of the aromatic protons rather than the aliphatic ones. Obviously, the chemical shift changes towards the higher field associated with decrement of signal intensity, and indicates that the H-bonding is weakened with the temperature as expected, and this provides evidence that this part of the molecule could be subjected to conformational changes under breaking of the H-bonding. One of the signals that is highly affected under the temperature gradient is the O-H group of the COOH of the carboxylic acid, *δ* = 12.59. Increment the temperature of the DMSO solution lead to higher magnetic field for the resonance with the decreasing of the signal intensity. From such results of the FT-IR and NMR, it is possible to prove that H-bonding between the cyano derivative and the carboxylic acid occurs.

#### Mass Spectrometer and Chromatography confirmations:

**FIGURE 4:**
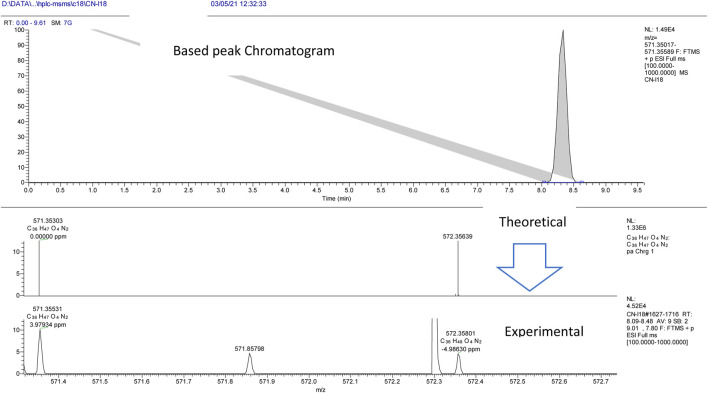
HPLC-MS extracted ion chromatogram of the compound entitled I/A8 at *m/z* 571.35531 (RT: 8.3 min).

**FIGURE 5:**
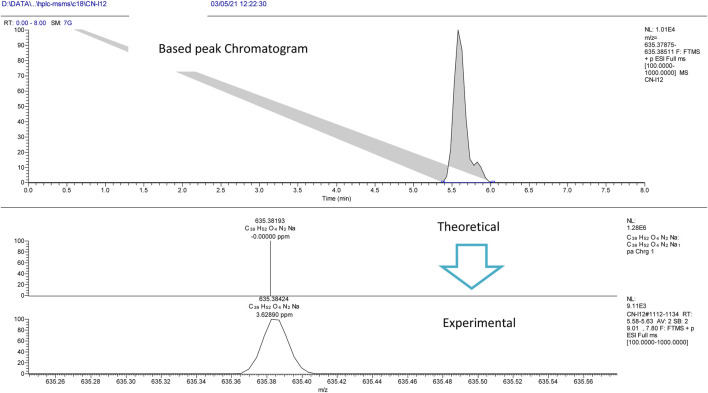
HPLC-MS extracted ion chromatogram of the compound entitled I/A12 at *m/z* 635.38424 (RT: 5.7 min).

The experiment was performed with the OrbitrapID-X mass spectrometer (Thermo Scientific). The Orbitrap IDX spectrometer could reach a high resolution (> 120,000) and reliable mass accuracy (< 5 ppm mass error). The mass scan range was set to 100–2000 m/z and the Electrospray ionization was set in positive mode (ESI+) for the studied compounds. The Mass spectrometer was calibrated using a purchasable “Calibration Mix ESI (Thermo Scientific)” by following the manufacturer guideline. The ESI was carried out using a heated ion source with a metal needle and a 3.5 kV voltage. The temperature of the source vaporizer was set to 350°C, the capillary temperature to 275°C, and the sheath and auxiliary gases were optimized to 40 and 20 arbitrary units, respectively. The samples were automatically infused (5 µL each) through the HPLC system (Vanquish, Thermo Scientific). The separation was performed with the use of column (Acquity UPLC HSS C18, 2.1 × 50°mm, 1.8°µ). A heated ion source with a metal needle and a 3.5 kV voltage were used in the ESI. The source vaporizer’s temperature was set to 350°C, the capillary’s to 275°C, and the sheath and auxiliary gases were set to 40 and 20 arbitrary units, respectively. The flow rate was set to 0.45 ml/min and a gradient was applied for the separation as follow. 0–1 min (95%A, 5%B); 1–7 min (5%A, 95%B); 7–9 min (5%A, 95%B); 9.1 min (5%A, 95%B); 10 min (5%A, 95%B). Xcalibur™ software (Thermo Scientific) was used for method development and data treatment. Representative examples of I/A8 and I/A12 complexes measurements are depicted in Figures 4 and 5, respectively.

### Mesomorphic and Optical Studies

**FIGURE 6:**
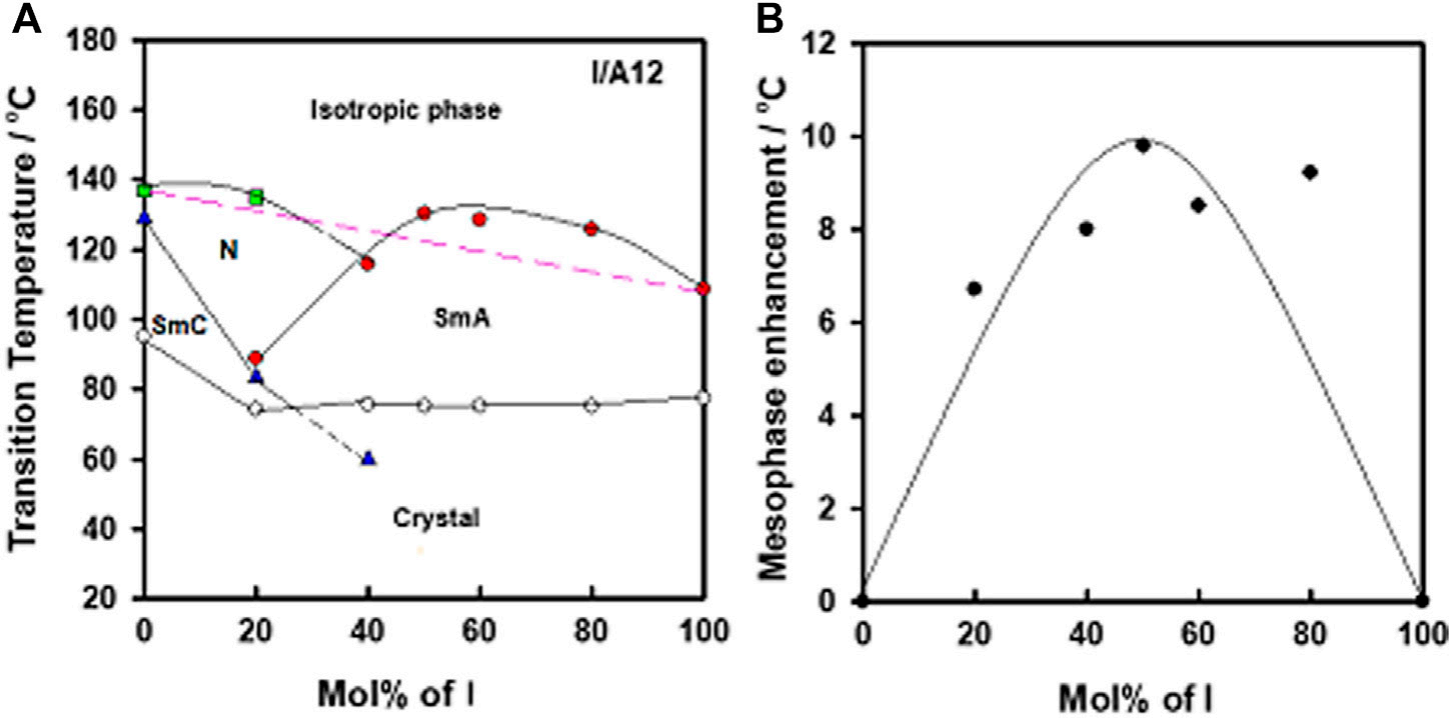
**(A)** Binary phase diagram of I/A12 SMHBC, and **(B)** the relation of the mesomorphic enhancement and the mixture compositions of base, I.

In order to confirm the complex formation between N-4-cyanobenzylidene-4-(hexyloxy)benzenamine (I) and alkoxy benzoic acids (An), a binary phase diagram was made for the system I/IA12 as an example. It was found that the difference between H-donors and H-acceptor in polarity affects the strength of the hydrogen bonding interactions (Cleland and Kreevoy, 1994). However, the polarities of both components of the mixture are not affected by the terminal length of attached chain. The graphical binary phase diagram is presented in Figure 6A. As can be seen, induced nematic and SmA mesophases are of higher thermal stabilities than that deduced from the linear-dependence (wide range ≈ 50% mol of I, see Figure 6). This arises from the minimum potential energy surface, which is used to describe the non-ionized H- bond between an acid and base components (Martinez-Felipe et al., 2016). Such mesomorphic enhancement (∆T = Tmeasured–Tlinear) is correlated with the mixture composition in Figure 6B. The 1:1 M supramolecular complex formation can be confirmed from the enhanced mesophase thermal stability at this composition (≈ 50% mol of I), (Martinez-Felipe et al., 2016; Ahmed et al., 2019a).

**FIGURE 7:**
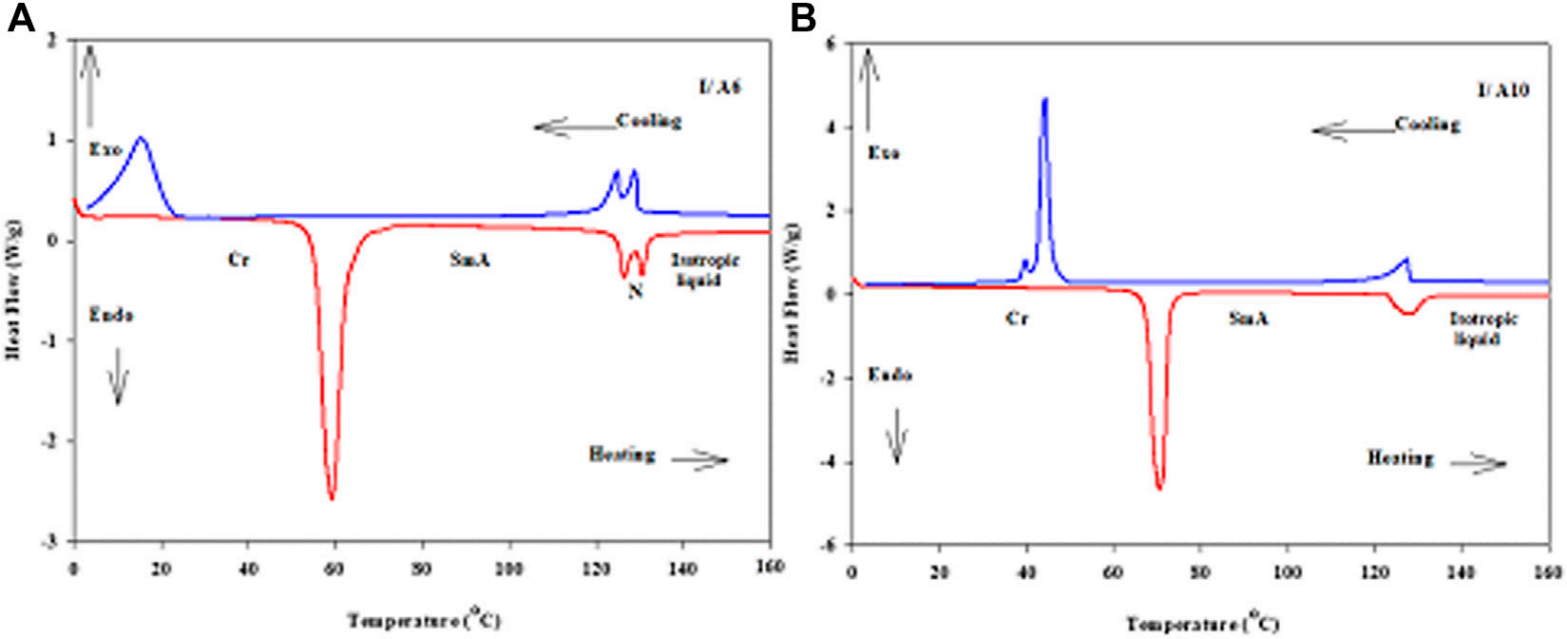
DSC thermograms at a heating rate 10^o^C/min for heating and cooling scans of 1:1 SMHBCs **(A)** I/A6 and **(B)** I/A10.

Continuing the confirmation, 1:1 M ratios of supramolecular complexes, I/An, made from CN derivative as a base component, I, and each of the five acid homologues, An, were prepared and investigated for their mesomorphic activities via DSC and POM. DSC analyses were confirmed by the POM texture investigations. In order to ensure the thermal stability of the mixtures, the DSC analyses were performed for two heating–cooling scans. All thermal investigations of present SMHBCs were recorded from the second heating scan, and the DSC thermogams (Figure 7) possess similar behaviors and reveal that the prepared mixtures are very clean. The DSC thermograms of other prepared complexes are depicted in Supplementary Figures S1–S3 (supplementary data). DSC thermogram of I/A6 showed three endotherms characteristic peaks of the crystal–SmA, SmA-N and N–isotropic transitions upon heating and reversed upon cooling scan. While, thermograms of I/A8, I/A10, and I/A12 showed two endothermic peaks of the crystal–SmA and SmA–isotropic liquid phase transitions upon heating and reversed also on cooling. The DSC curve of the longest chain complex I/A16 exhibits three endothermic characteristics of the crystal–SmC, SmC-SmA and SmA–isotropic transitions upon heating and reversed upon cooling curve. Moreover, all melting transitions of samples are shifted to lower temperatures compared with those observed through heating cycle.

**FIGURE 8:**
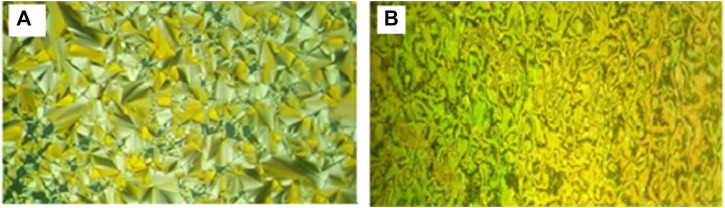
Textures upon heating as observed under POM for SMHBC I/A6: **(A)** SmA phase 98.0°C; **(B)** Nematic phase at 128.0°C.

Representative examples of POM textures are represented in Figure 8, which shows images of SmA and nematic mesophases of the dimorphic complex I/A6. In addition to, the presence of conic focal texture indicates the occurrence of the SmA phase, and the Schlieren texture means nematic phase.

It is important to mention that the pure 4-n-alkoxybenzoic acids exhibit smectic C and nematic mesophases dependent on their terminal length (Naoum et al., 2010), while the cyano derivative, I, possesses a smectic A phase with mesomorphic thermal stability 108.6^o^C (Nafee et al., 2020b)

**FIGURE 9:**
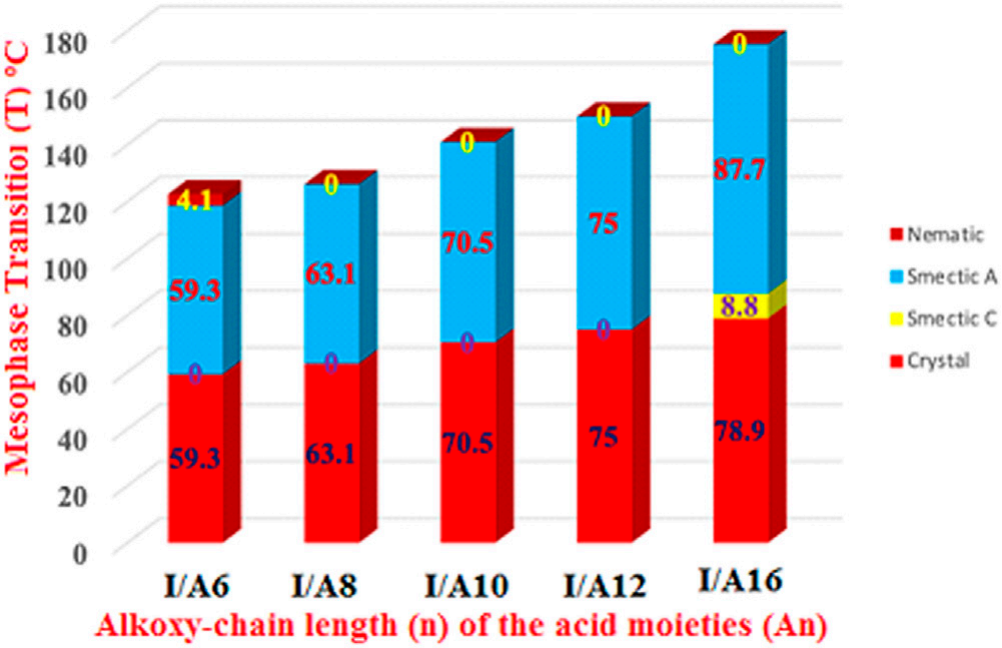
Effect of the terminal length of alkoxy-acid chain (n) on mesomorphic behaviour of 1:1 SMHBCs, I/An.

The mesomorphic and optical behaviors of the 1:1 SMHBCs (I/An) were analyzed. Transition temperatures (T) and their associated enthalpy (∆H) of mesomorphic transition, as derived from DSC investigations, for all prepared SMHBCs are tabulated in Table 1. Figure 9 displays the graphical relation of the chain-length and mesomorphic transition temperatures of the designed complexes in order to evaluate the effect of the terminal length of acid on the mesophase properties. It can be seen from Table 1 and Figure 9 that the enantiotropic mesophases are exhibited by all formed complexes, and their types are dependent on the length of the alkoxy chain n. On the other hand, the melting points of the investigated SMHBCs are only slightly affected by the length of the acid component chain (n). The higher melting temperatures of I/A16 complex attests to the increased amount of co-linearity of this mixture, which facilitates more efficient packing within the crystal phase and influences molecular interactions arising from the azomethine linker. In addition, the stability of the smectic A is also impacted by the incremental changes in the length of the acid chain (Naoum et al., 2010).

**TABLE 1:** Mesomorphic transition temperatures (°C), enthalpy (kJ/mol), and normalized entropy of transitions for present SMHBCs I/An.

System	*T*_Cr-SmC_	*T*_SmC-SmA_	*T*_Cr-SmA_	*T*_SmA-N_	*T*_SmA-I_	*T*_N-I_	*∆S/R*
**I**	-	-	77.3 (30.27)	-	108.6 (2.36)	-	0.74
**I/A6**	-	-	59.3 (49.17)	126.4 (7.87)	-	130.5 (3.12)	0.93
**I/A8**	-	-	63.1 (62.27)	-	131.5 (9.85)	-	2.92
**I/A10**	-	-	70.5 (70.08)	-	127.9 (9.79)	-	2.94
**I/A12**	-	-	75.0 (72.95)	-	130.4 (10.59)	-	3.16
**I/A16**	78.9 (85.30)	87.7 (3.85)	-	-	125.6 (11.18)	-	3.37

Cr-SmC = solid to the SmC phase transition.

SmC-SmA = SmC to the SmA phase transition.

Cr-SmA = solid to the SmA phase transition.

SmA-N = SmA to the N phase transition.

SmA-I = SmA to the isotropic phase transition.

N-I = nematic to the isotropic phase transition.

***ΔS***/R = normalized entropy of transition.

In the case of the shortest complex I/A6, it is dimorphic exhibiting both SmA and N mesophases with a thermal mesomorphic stability of 130.5^o^C and a mesomorphic range of 71.2^o^C. For the complexes, I/A8, I/A10, and I/A12, only the monomorphic SmA phase is observed, with thermal stabilities of 131.5, 127.9, and 130.4^o^C, respectively. For the longest acid chains (*n* = 16), complex I/A16 exhibits dimorphic mesophases, SmC (range ≈ 8.8^o^C) and SmA phases. In conclusion, the length of the terminal alkoxy chain on the acid component plays an important role and is more influential in the stability of the observed mesophases. The mesomorphic temperature range of the present SMHBCs are decreasing in the order I/A6 > I/A8 > I/A10 > I/A16, i.e., with linear dependency on the chain length (n). It has been reported that as the terminal chain length increases, the rigidity of SMHB central core will be decrease, and consequently, the structural linearity of the complex slightly decreases due to the large number of the chain configurations that result in strong interactions between the terminals (Ahmed et al., 2019b).

### Molecular Geometries Studies

**FIGURE 10:**
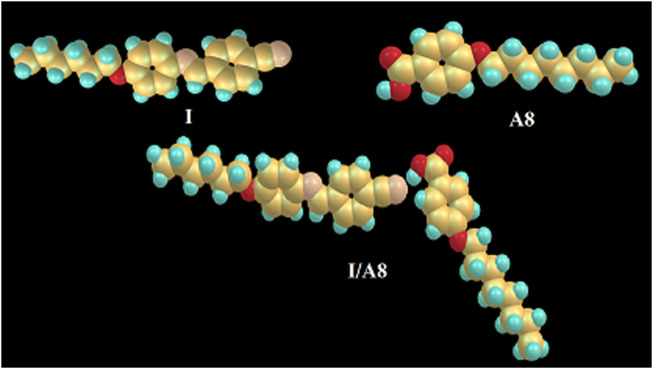
The calculated molecular geometry of the cyano derivative I, 4-octyloxybenzoic acid A8 and their SMHBC, I/A8.

The molecular geometry of the prepared SMHBCs (I/An) has been estimated using DFT calculations by the DFT method at basis set B3LYP 6-31G (d,p). All structures were minimized and optimized by the guesstimate of the geometrical optimization for each molecule to find the geometrical structure of minimum energy. The optimization process has proceeded to find the conformations of the minimum energy geometrical structure, where, the atoms, the bond lengths and bond angle of the molecules varied to find a new minimum energy geometry which is called as convergence. The fact that imaginary frequencies are not present is evidence of the geometrical stability of all H-bonded complexes. Figure 10 shows the optimum geometrical structure of the cyano derivative (I) and 4-octyloxybenzoic acid A8 as well as their H-bonded complex I/A8.

Although both the individual components of the cyano derivative I and 4-alkoxybenzoic acids are linear and have complete planar geometry, the SMHBCs derivatives are non-linear, as shown in Figure 10, the supramolecular complexes I/An exhibiting a non-linear, gun-shaped geometry. It is possible to take the absent imaginary frequencies as evidence for their geometric stability. The results of the current calculations tell us the preferred molecular geometry in gas phase; however, the condensed mesophases have liquid crystalline present, meaning that the lowest energy may differ, making the elongated species the more preferred geometry.

**FIGURE 11:**
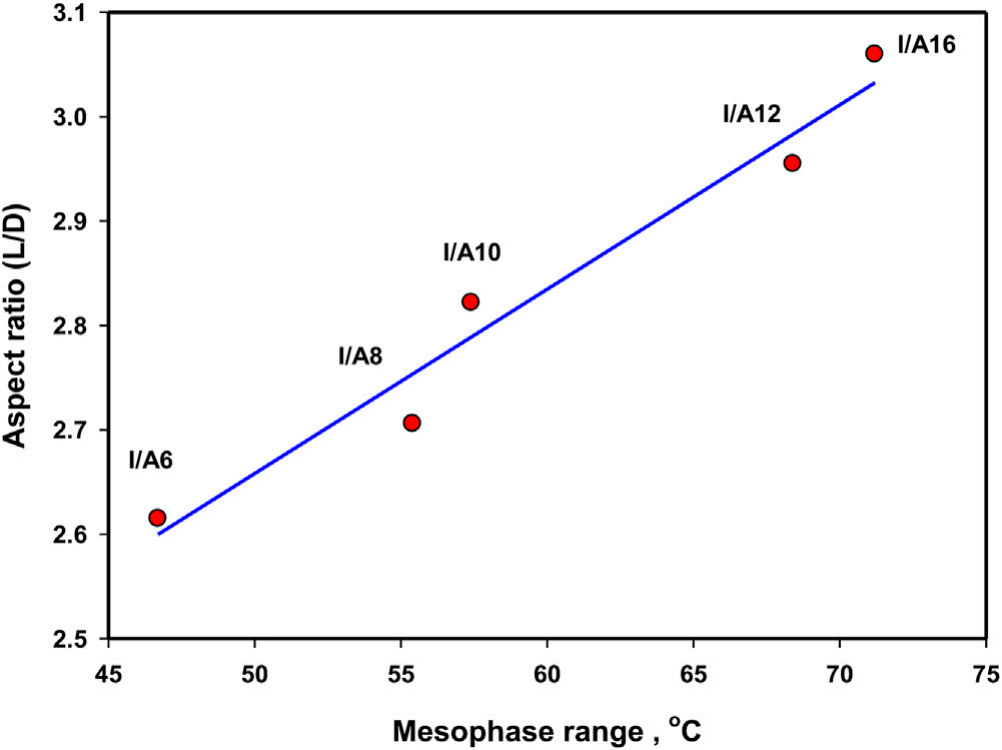
Dependence of the mesophase range with aspect ratio of SMHBCs, I/An.

In order to investigate the impact of the chain length on the mesomorphic behavior of the SMHBCs, the aspect ratios (L/D, see Table 2) were calculated by estimating the dimension parameters, L = length and D = width. Due to the non-linear geometry of the SMHBCs, the dimensional parameters increases by small values as the length of the alkoxy terminal of the carboxylic acid increases. Moreover, the increment of the length of the complexes is less than that of the width, and consequently the aspect ratios decrease with the terminal alkoxy chain length. As shown in Figure 11, the mesophase range decreases with the aspect ratio. This result can be illustrated in terms of the decrement of the lateral interaction with longer chain length due to the lower aspect ratio. The higher the aspect ratio, the more the molecules can pack together. Moreover, the higher aspect ratios of the SMHBCs I/An at longer lengths of the terminal chains could explain the formation of the nematic mesophase. The higher aspect ratios at shorter chain lengths resulted in the decrement of the lateral interaction, allowing the terminal interaction to be dominant to enhance the less ordered nematic phase. However, the longer terminal chains produce a dilution of the aromatic moieties with lowering aspect ratios to enhance side-side attraction over end-end aggregation, and consequently the more ordered smectic mesophase is observed.

**TABLE 2:** Dimensional parameters and aspect ratios of SMHBCs, I/An.

Parameter	I/A6	I/A8	I/A10	I/A12	I/A16	
Dimensions Å	Length (L)	37.790	39.858	41.766	43.409	47.840
	Width (D)	12.350	13.487	14.802	16.045	18.292
Aspect ratio (L/D)	3.060	2.955	2.822	2.706	2.615	
Mesophase range	71.2	68.4	57.4	55.4	46.7	

**FIGURE 12:**
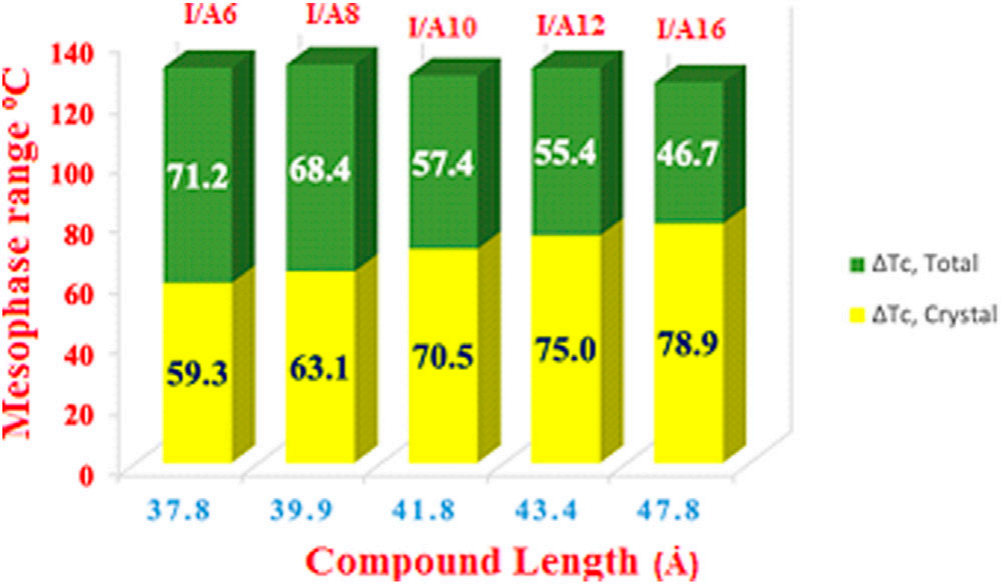
Dependence of the mesophase range on the length (Ȧ) of SMHBCs, I/An.

An important element that could affect the mesophase range of the angular shaped liquid crystals is the width of the compounds. Figure 12 shows the relationship between the calculated width of the prepared supramolecular H-bonded complexes and the enhanced mesophase range. Notably, as the length of the alkoxy terminal chain increases, the width of the angular complex increases. The successive increment of the crystal mesophase and the decrement of the other enhanced mesophase ranges. This could be explained by the higher degree of the intermolecular interaction with higher chain lengths. At higher chain lengths, the van der Waal’s interaction increases to enhance the formation of the highest ordered crystal mesophase due to the enforced parallel association.

### Thermal Parameters

**FIGURE 13:**
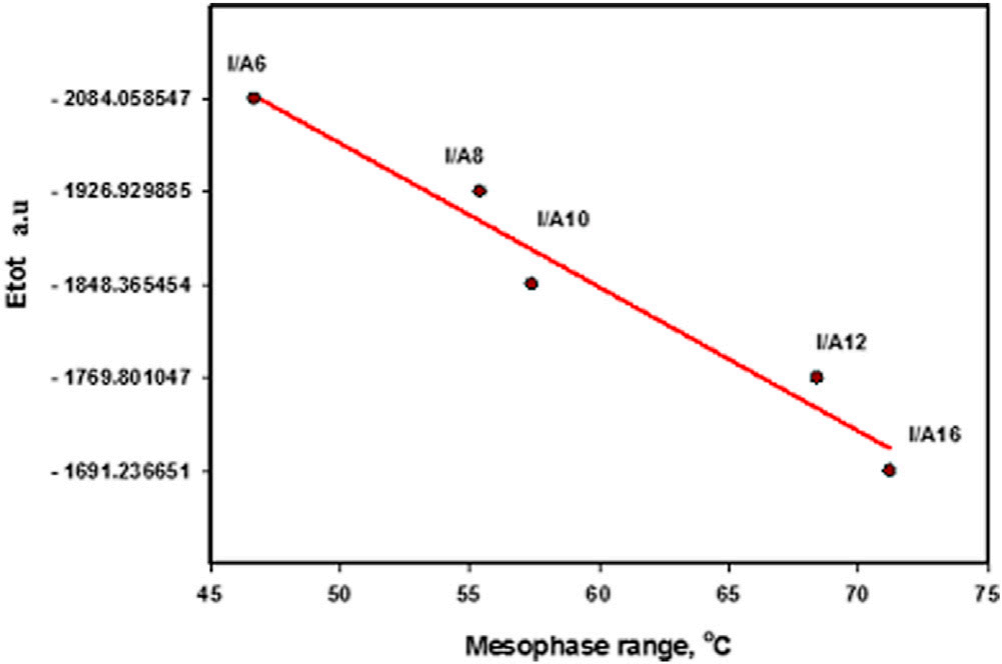
Relationship between the sum of the electronic and thermal energies with the smectic mesophase stability of H-bonded complexes, I/An.

The estimated thermal parameters were calculated with the same method at the same set for the cyano derivative as well as its H-bonded complexes (I/An), and the data are collected in Table 3. Obviously, the calculated total predicted stability of the SMHBCs increases with increasing chain length. This could be explained by the incremental molecular packing at higher chain lengths, resulting in lower energy. The longer the chain length, the more the alkoxy chains aggregated due to Van der Waals forces, and the lower the predicted energy values of SMHBCs. As shown in Figure 13, longer alkoxy chains result in more stable SMHBCs (I/An), and have a negative effect on the smectic mesophase range. The decrement of the smectic range can by explained by the terminal lengths; as the chain length increases, the strength of the terminal aggregation increases, resulting in the decrease of the smectic range.

**TABLE 3:** Thermal parameters (Hartree/Particle) of the H-bonded complexes I/An.

Parameter	I	I/A6	I/A8	I/A10	I/A12	I/A16
Ecorr	0.376839	0.667563	0.724793	0.781819	0.838814	0.953074
ZPVE	−959.673700	−1691.277723	−1769.844771	−1848.411939	−1926.979094	−2084.113191
Etot	−959.651417	−1691.236651	−1769.801047	−1848.365454	−1926.929885	−2084.058547
H	−959.650473	−1691.235707	−1769.800103	−1848.364510	−1926.928941	−2084.057603
G	−959.729290	−1691.364803	−1769.934774	−1848.506657	−1927.078202	−2084.220908

ZPVE: Sum of electronic and zero-point energies; Etot: Sum of electronic and thermal energies; H: Sum of electronic and thermal enthalpies; G: Sum of electronic and thermal free energies.

### Frontier Molecular Orbitals and Polarizability

**FIGURE 14:**
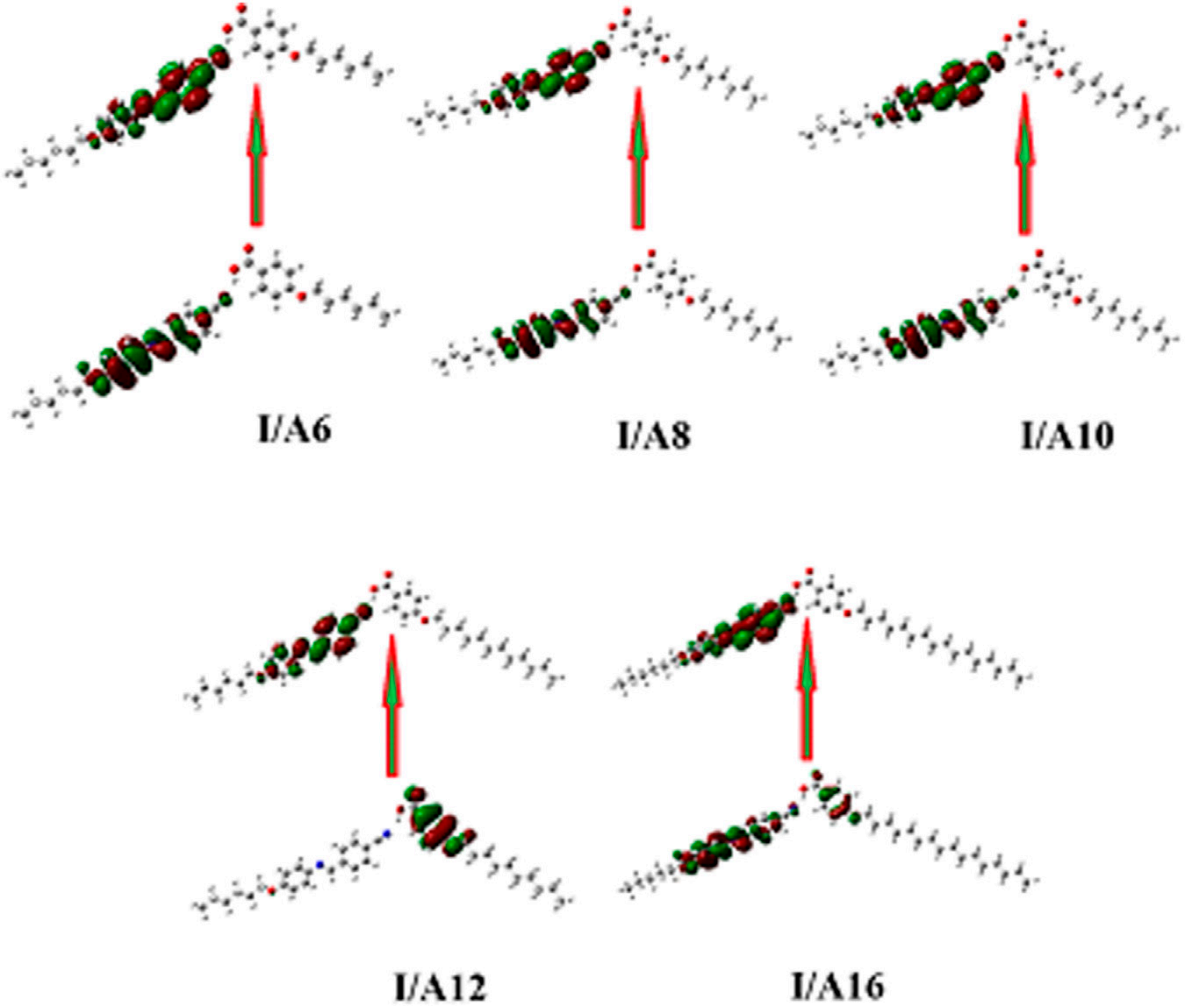
The estimated plots for Frontier molecular orbitals of SMHBCs, I/An.

Figure 14 depicts the approximate plots of the prepared SMHBCs **I/An** for Frontier molecular orbitals, FMOs, HOMO (highest occupied molecular orbitals), and LUMO (lowest unoccupied molecular orbitals). The cyano derivative is clearly where the atom electron densities involved in FMO formation. Further, as shown in Table 4, the alkoxy chain length has no significant effect FMO energy levels, or the gaps of energy between HOMO and LUMO. The energy gap between FMOs could be to predict the efficiency and likelihood of electron transfer between the FMOs during a electronic excitation process. Moreover, it can be used to calculate parameters such as the global softness (S = 1/ΔE) and chemical hardness η: parameters that depend on polarizability and liquid crystal sensitivity for photoelectric effects. The better global softness of the compounds leads to greater enhancement of their polarizability as well as the photoelectric sensitivity. As shown in Table 4, the H-bonded complexes derived from the cyano compound I have a lower FMO energy gap than does the individual cyano compound; consequently, formers are softer than that of later. Moreover, the H-bonding of the cyano liquid crystal I increases the polarizability by almost 100 units from 284 to 465–584 Bohr^3^. The higher polarizability can be explained in terms of the higher aspect ratio of the SMHBCs with respect to the individual components. Moreover, the more diminished the energy difference of I/An, the more their polarizability increases.

**TABLE 4:** FMO Energies a.u., Polarizability, α, and Dipole Moment *μ* (Debye) of the cyano compound **I** and its SMHBCs, I/An.

Parameter	I	I/A6	I/A8	I/A10	I/A12	I/A16
ELUMO	−0.08971	−0.10132	−0.10140	−0.10158	−0.10159	−0.10157
EHOMO	−0.22201	−0.22882	−0.22877	−0.22886	−0.22889	−0.22882
ΔEHOMO-LUMO	0.1323	0.1275	0.1274	0.1273	0.1273	0.1273
Softness, S	7.559	7.843	7.849	7.855	7.855	7.855
Hardness, η	0.0662	0.0638	0.0637	0.0637	0.0637	0.0637
μ Total	6.707	9.902	10.988	10.981	10.977	10.973
Polarizability α	284.89	465.43	488.94	512.43	535.00	584.04

However, the dipole moment investigation is one of the most important parameters that can impact enhanced mesophase type and behavior. From Table 4, it is clear that the dipole moment of H-bonded complexes I/An is higher than that of the cyano liquid crystal, 9.9-10.9, 6.7 Debye, respectively. The higher dipole moment of the H-complexes explains well the smectic texture of higher range of stability of the formed mesophase. The higher dipole moment is predominant in side-side interactions rather than end-end interactions by permitting the lateral stacking to be dominant to enhance the smectic phase, 29.2^o^C mesophase range of the cyano LC (I) and 71.2^o^C for I/A6.

**FIGURE 15:**
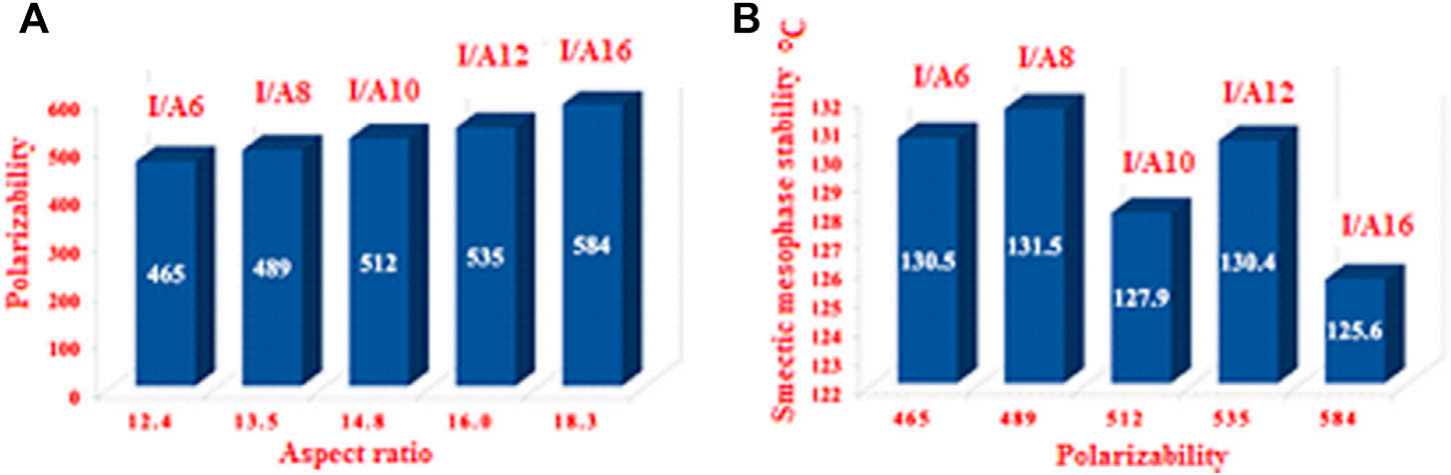
The relationship between the polarizability and the aspect ratio (L/D) of all prepared compounds **(A)**; and polarizability dependence relationship with the length of alkoxy chains **(B)**.

It is well known that changing the dimensional parameters as well as the polarity of the terminal attached substituents of the liquid crystals has a major effect on the polarizability (Kim and Kastelic, 1984; Sengupta et al., 2013). As shown in Figure 15 **,** as the alkoxy chain length increases, the polarizability increases, this can be explained in terms of the aspect ratio. As the aspect ratios of the molecule increases, the space filling of the liquid crystalline compounds increases, resulting in the enhancement of the polarizability. On the other hand, the increment of the polarizability irregularly affects the smectic mesophase stability. However, as the aspect ratio increases, the complexity of the materials increases, and so the lateral interaction as well as the van der Waal’s intermolecular interaction increase. The higher degree of the intermolecular forces permits the enhancement of the more ordered smectic mesophases.

### Molecular Electrostatic Potential

**FIGURE 16:**
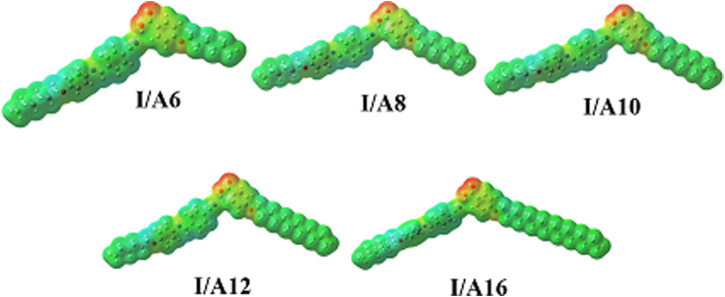
Molecular electrostatic potentials (MEP) for SMHBCs I/An.

According to the molecular electrostatic potential, the charge distribution map for SMHBCs of SMHBCs **I/An** was determined using the same method on the same basis sets (MEP) (Figure 16). The negatively charged atomic sites (the red region) were thought to be mostly concentrated on the alkoxy acid hydrogen bonded carboxylate moiety. The alkyl chain and the cyano derivative moiety are expected to have the least negatively charged atomic sites (blue regions). As shown in Figure 16, the orientation and magnitude of charge are heavily influenced by H-bonding. As a result, this may be used to understand why the dipole moment of the SMHBCs increases when compared to the cyano LC. The SMHBCs **I/An** longer alkoxy chain lengths have no impact on charge orientation or magnitude.

### Entropy Change of SMHBCs

From the entropic changes perspective, flexible chains dominate since they are very labile and can easily form multi-conformational modes. Entropy change is observed in all formed 1:1 mixtures, more conformational and orientational changes in the mixture as compared to the individual, pure compounds.

**FIGURE 17:**
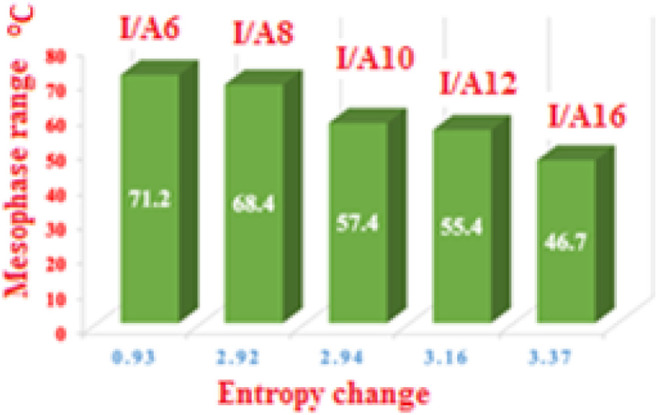
Entropy changes as a function of the terminal alkoxy chain length and the mesophase range.

The normalized entropy of the clearing transitions (∆S/R) were calculated from DSC measurements for all investigated SMHBCs, I/An, and the data are tabulated in Table 1. Results indicate a linear correlation linear variation between the entropy change and the length of the terminal flexible-chains on the acid moiety (see Figure 17). This may be attributed to differences in molecular interaction enhancements as the chains increase. As the chain length increases, the more ordered smectic phase observed, and results in the high difference in the entropy change between the smectic to the isotropic phases. Although, the longer chain length decreases the mesophase range, it increases the smectic entropy change; this finding can be explained in terms of the enhanced crystal mesophase with the longer chain length.

## Conclusion

A new series of SMHBCs were formed from 4-n-alkoxybenzoic acid (An) and N-4-cyanobenzylidene-4-(hexyloxy)benzenamine (I). Several points have been concluded from this investigation:

•FT-IR, NMR temperature dependency, as well as binary mixtures systems were proved the SMHBCs formation.
•The temperature gradient NMR spectroscopy showed a shift in the aromatic protons rather the aliphatic one and the O-H signal was diminished with high temperature as well as it is shifted to higher field.
•Mesomorphic and optical characterizations results showed that enantiotropic N and SmA mesophases.
•The mesomorphic temperature ranges decreased in the order I/A6 > I/A8 > I/A10 > I/A16 with linear dependency on the terminal chain length.
•The DFT calculations showed that a non-linear, gun-shaped geometry of the complexes I/An.
•The mesophase range decreases with the calculated aspect ratio.
•The decrement of the smectic range has been explained in terms of the effect of the terminal lengths impact.
•Linear variation in the entropy change was observed with the terminal flexible-chain length of the acid moiety.

## Data Availability

The original contributions presented in the study are included in the article/Supplementary Material, further inquiries can be directed to the corresponding authors.
