# Supplementary material for: New Liquid Crystal Assemblies Based on Cyano-Hydrogen Bonding Interactions
Source: Front Chem. 2021 Jun 4;9:679885. doi: 10.3389/fchem.2021.679885 (PMC8213374; doi:10.3389/fchem.2021.679885)
Supplement: Supplementary file 1 [file DataSheet1.docx]

**Supplementary Data**

New liquid crystal assemblies based on cyano-hydrogen bonding interactions

Mohamed Hagar^*1,2^, Rua B. Alnoman^1^, Mariusz Jaremko ^3^ , Abdul-Hamid Emwas ^4^, Salim Sioud^5^ , O.A. Alhaddad ^6^ and H. A. Ahmed^*7^

1. College of Sciences, Chemistry Department, Yanbu, Taibah University, Yanbu 30799, Saudi Arabia
2. Faculty of Science, Chemistry Department, Alexandria University, Alexandria 21321, Egypt
3. King Abdullah University of Science and Technology (KAUST), Biological and Environmental Sciences & Engineering Division (BESE), Thuwal, 23955-6900, Saudi Arabia.
4. King Abdullah University of Science and Technology. Core Labs. Thuwal 23955-6900, Kingdom of Saudi Arabia.
5. King Abdullah University of Science and Technology. Analytical Chemistry Core Lab. Thuwal 23955-6900, Kingdom of Saudi Arabia.
6. College of Sciences, Chemistry Department, Madina Monawara, Taibah University, Al-Madina 30002, Saudi Arabia. [alhaddad105@yahoo.com](mailto:alhaddad105@yahoo.com) (O.A.A.)
7. Department of Chemistry, Faculty of Science, Cairo University, Cairo 12613, Egypt.

*** C**orrespondence: M. Hagar, mohamedhaggar@gmail.com; H.A. Ahmed, ahoda@sci.cu.edu.eg

**H^1^-NMR supramolecular *H*-bonded complexes (I/A8)**

The NMR have been recoded for compound **I/A*8*** that was prepared by dissolving in 600 µl of deuterated solvents DMSO-d6 inside a 3ml glass vial, then vigorously vortexed until completely dissolved. 500 μl was transferred to 5 mm NMR tubes. A Bruker 600 NMR spectrometer (Bruker BioSpin, Rheinstetten, Germany) operating at 600.13 MHz for proton equipped with a triple resonance probe was used to record all NMR spectra. The ^1^H NMR spectrum was recorded by collecting 64 scans with a recycle delay time of 10 s, using one pulse sequence through a standard (zg) program from the Bruker pulse library. The ^13^C NMR spectra were recoded using the reported methods and parameters. Chemical shifts were corrected using the TMS signal at 0.0 pp as an internal chemical shift.


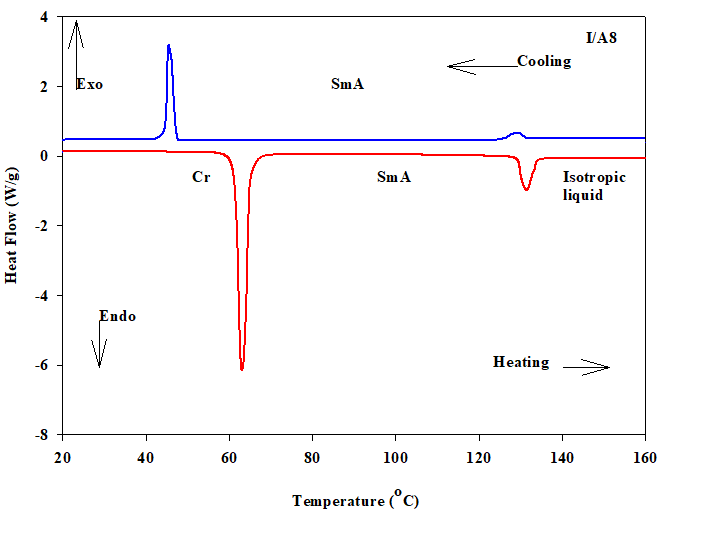


**Figure S1.** DSC thermograms of 1:1 SMHBCs of I/A8 at a heating rate 10 ^o^C/min for heating and cooling scans.


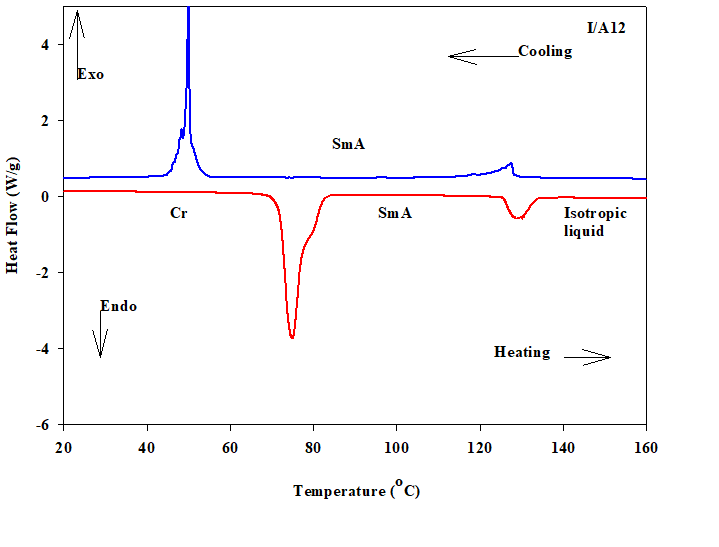


**Figure S2.** DSC thermograms of 1:1 SMHBCs of I/A12 at a heating rate 10 ^o^C/min for heating and cooling scans.


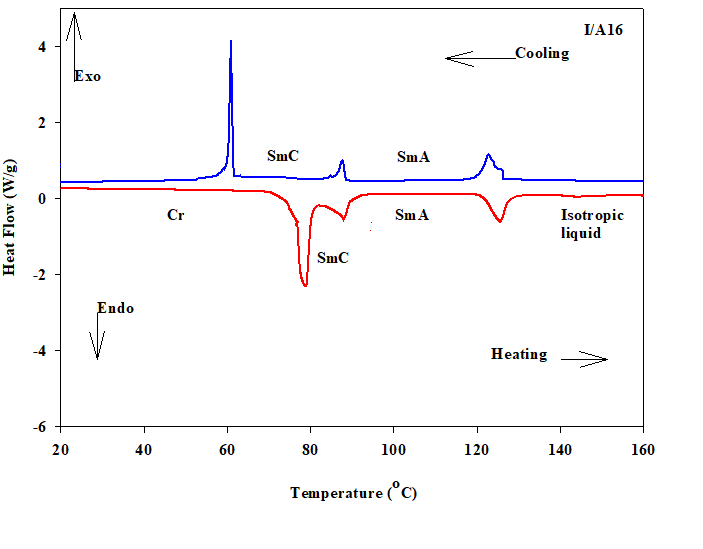


**Figure S3.** DSC thermograms of 1:1 SMHBCs of I/A16 at a heating rate 10 ^o^C/min for heating and cooling scans.


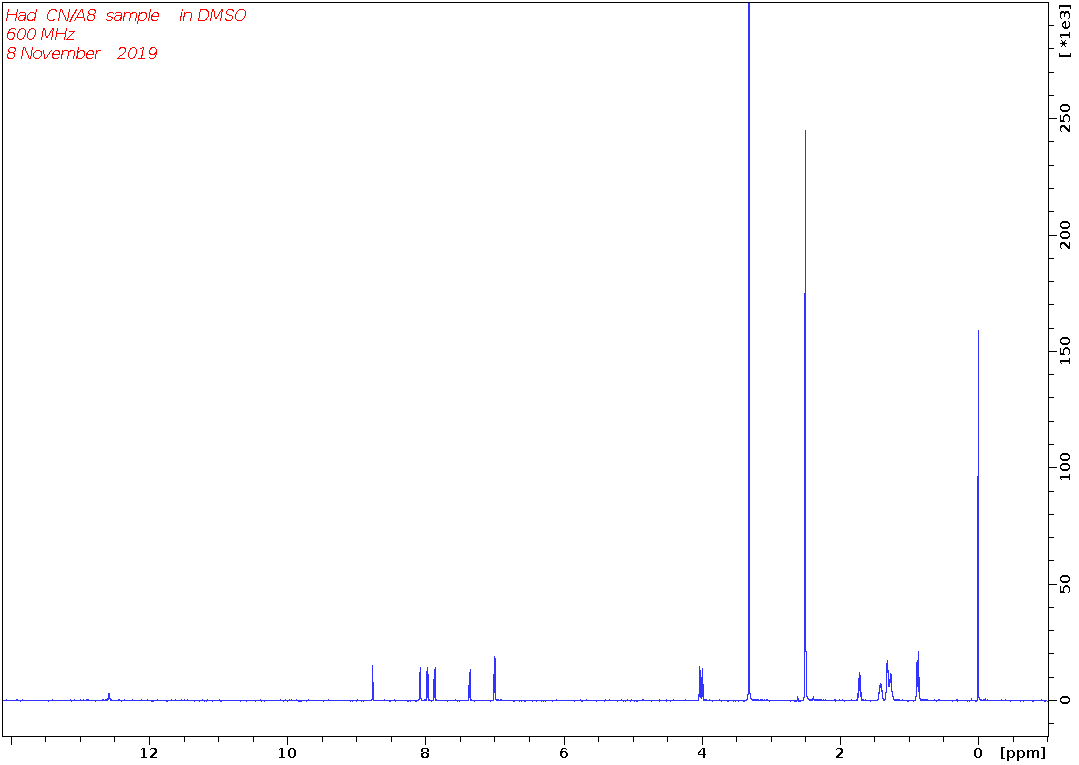


H^1^-NMR 1:1 supramolecular *H*-bonded complexes (**I/A8**) at room temperature.


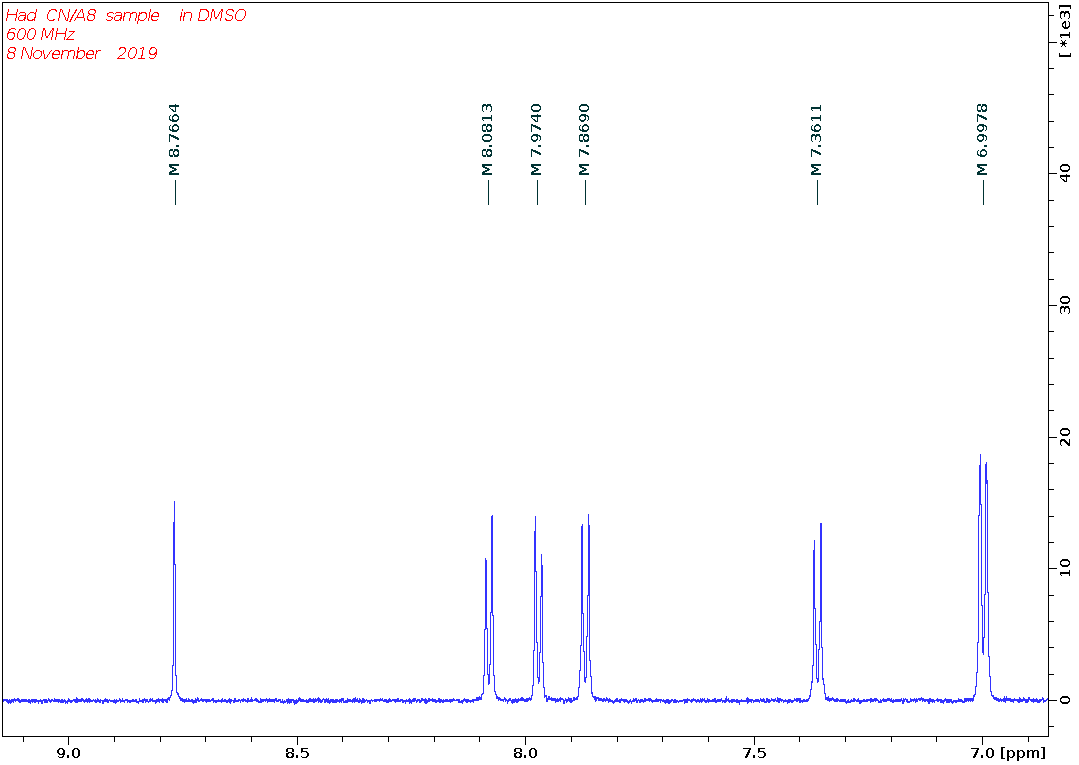


H^1^-NMR 1:1 supramolecular *H*-bonded complexes (**I/A8**) at room temperature.


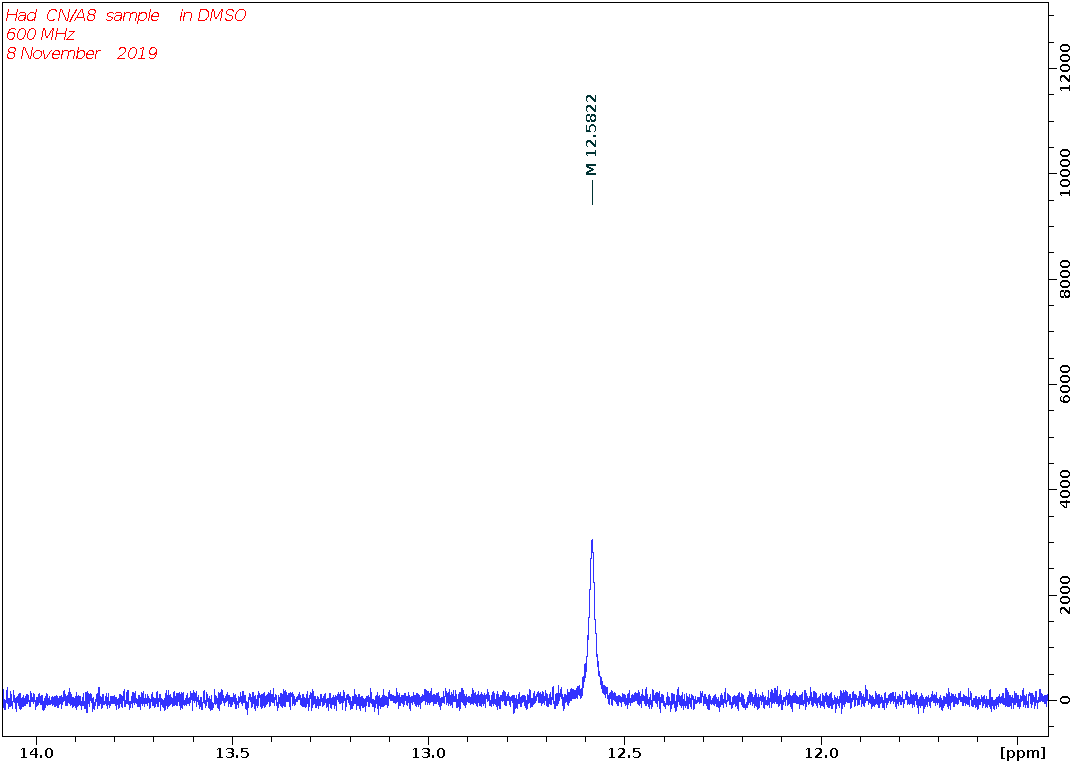


H^1^-NMR 1:1 supramolecular *H*-bonded complexes (**I/A8**) at room temperature.


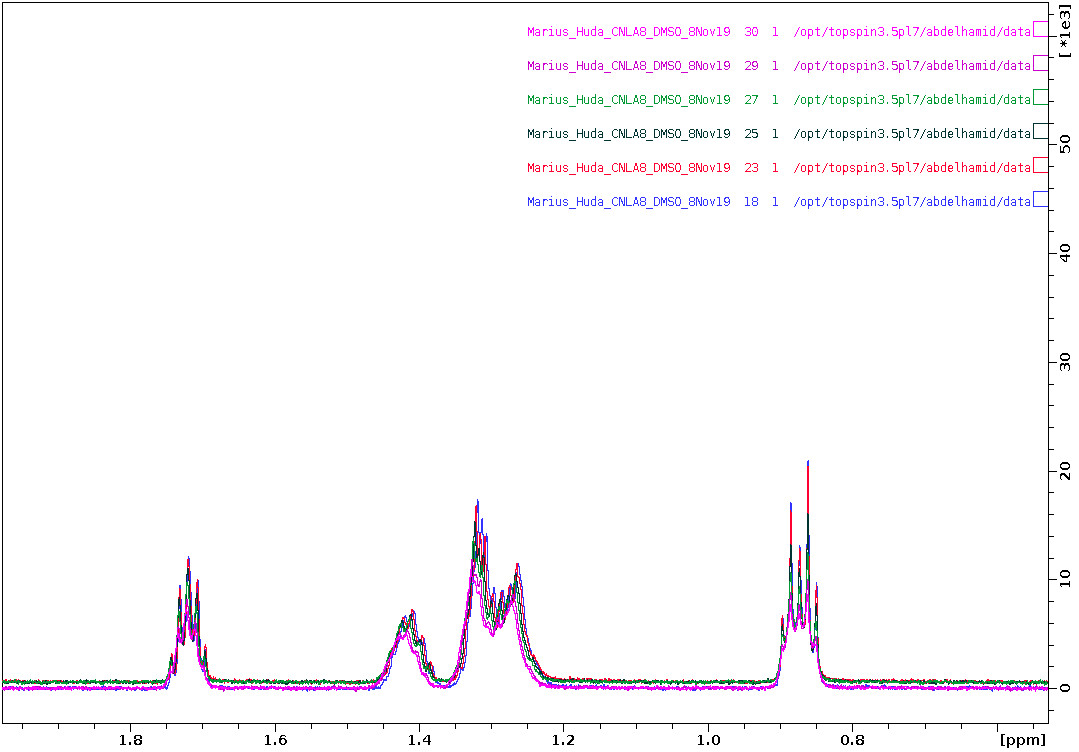


H^1^-NMR 1:1 supramolecular *H*-bonded complexes (**I/A8**) with temperature gradient.


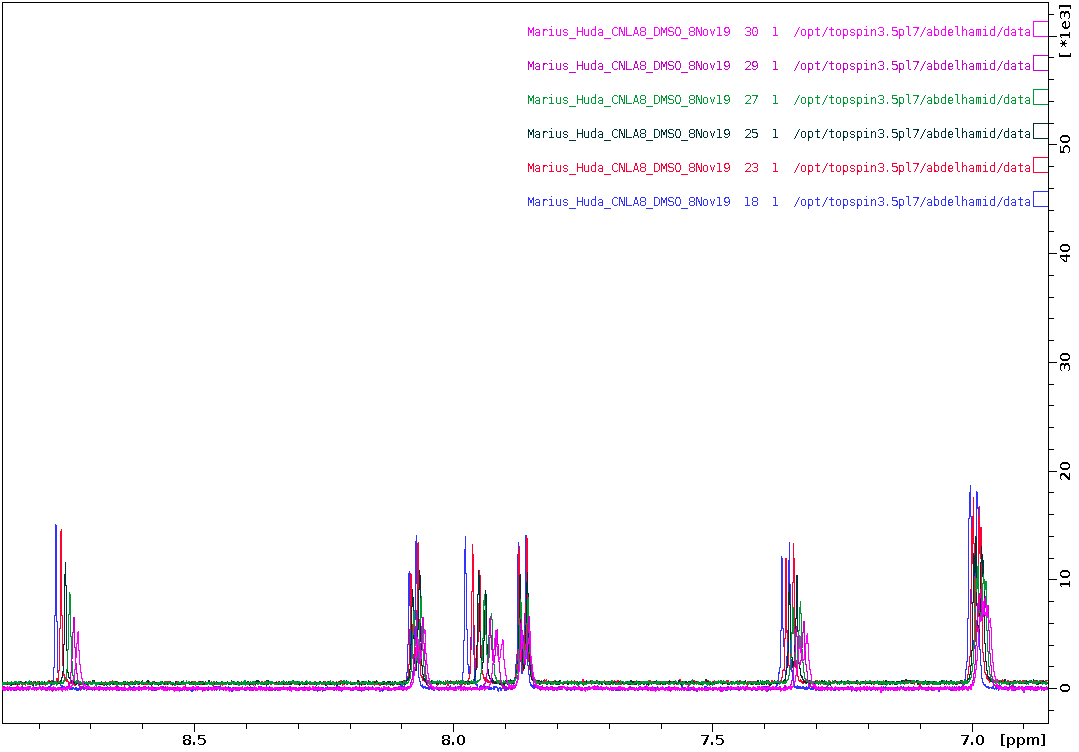


H^1^-NMR 1:1 supramolecular *H*-bonded complexes (**I/A8**) with temperature gradient.


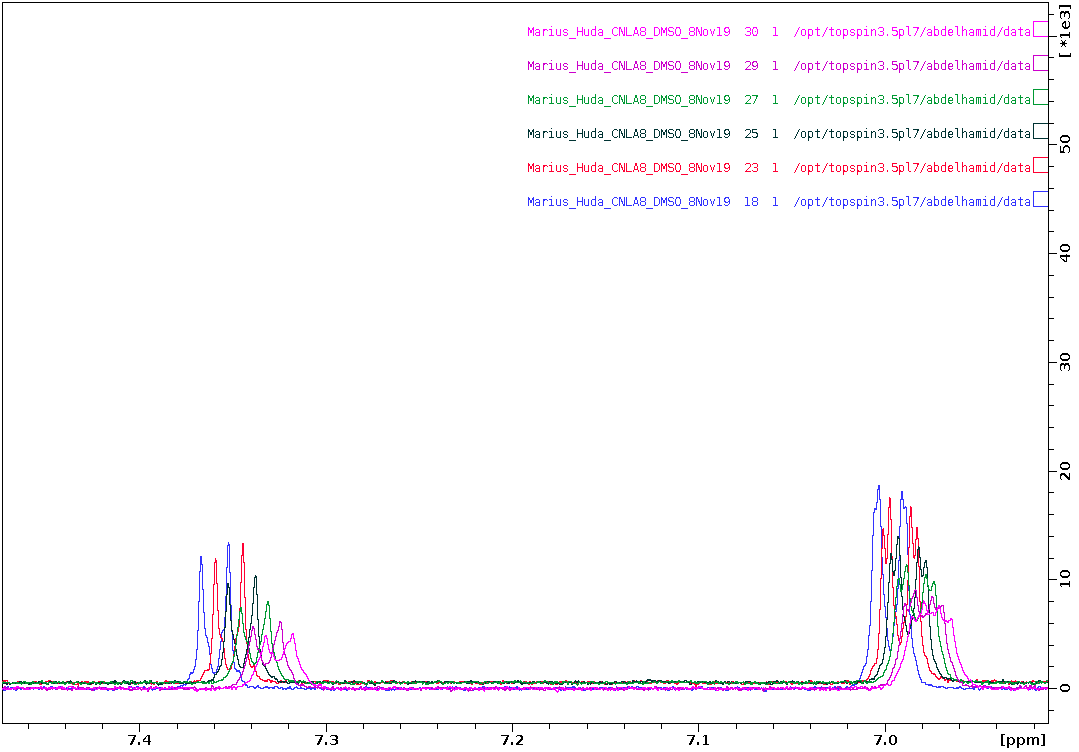


H^1^-NMR 1:1 supramolecular *H*-bonded complexes (**I/A8**) with temperature gradient.


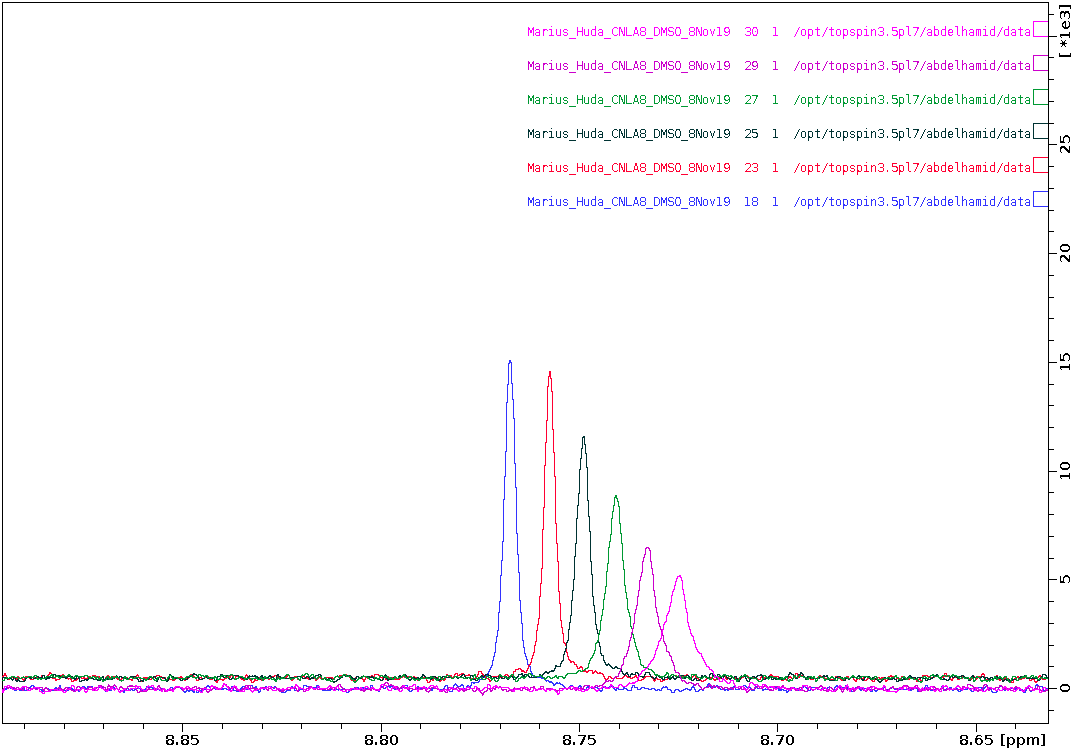


H^1^-NMR 1:1 supramolecular *H*-bonded complexes (**I/A8**) with temperature gradient.


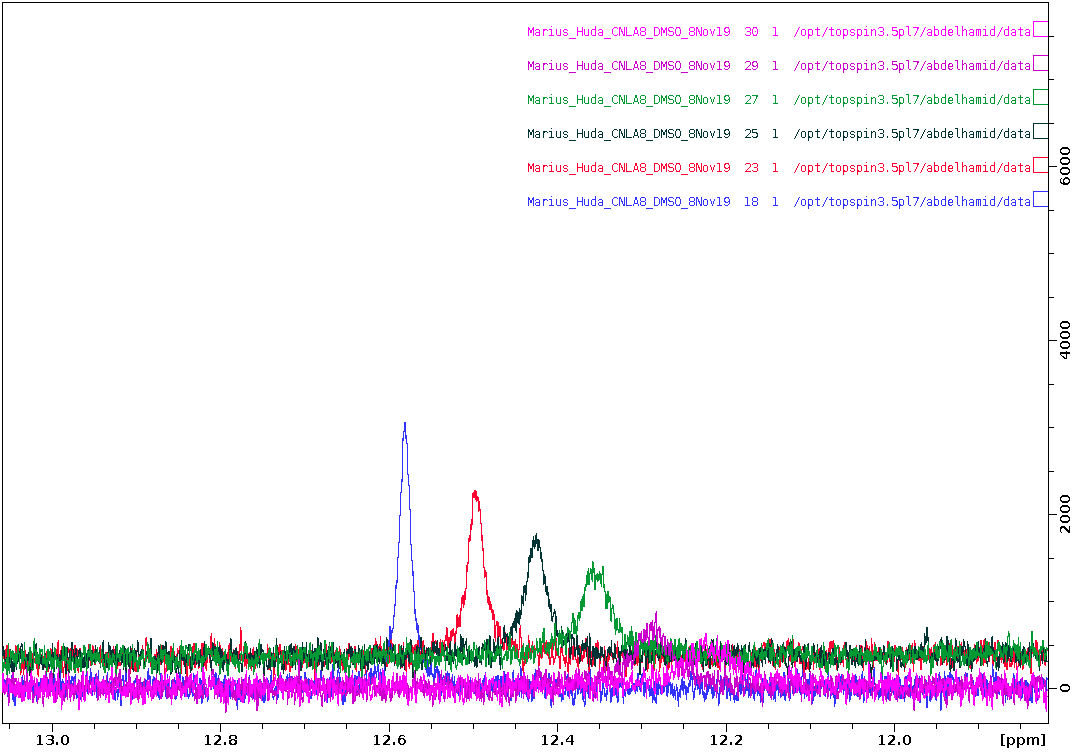


H^1^-NMR 1:1 supramolecular *H*-bonded complexes (**I/A8**) with temperature gradient.
